# The National Children Adoption Association

**Published:** 1921-05-14

**Authors:** 


### THE NATIONAL CHILDREN ADOPTION ASSOCIATION.

### BY A SOCIAL WORKER.

There is surely no human being more deserving of pity than an unwanted child. Considered merely as a national asset this condition gives the child less chance of survival, and very much less chance of developing to a healthy and happy maturity. Private charity in the past, followed by State and municipal effort, collected these forlorn babies into institutions, to discover, after generations of work and disappointment that the Almighty sets the poor in families and not in barrack organisations. Many have become the prey of the baby farmer; every now and then there has been some terrifying exposure of this hideous trade. Yet all the while there have been, and are, the childless homes, some in which animals are pampered pets, some in which a hunger of the heart goes unsatisfied.

A little quiet bit of work undertaken by Miss Clare Andrew at Exeter during the war for Belgian babies has sprung up like mustard-seed into an association for which, on Ascension Day, Princess Alice Countess of Athlone opened a second hostel or clearing-house, Tower Cressy, on Campden Hill, which holds some twenty children, being in full use. That this is so will surprise no one, but what is a constant source of amazed delight to the workers is that the stream of would-be adopters gathers volume as it flows. All sorts and conditions of people have come forward; at one moment two young teachers decide to live together and share a child; at another a childless couple in good social position ask for a long-clothes baby yearly until six shall be reached, which they hold to be the ideal number for a family; some other adopters are working-class people. Yet the rules are stringent. Only healthy children are accepted by the Association. When selection has been made, the child must be taken for a month on trial, an outfit being supplied by the adopter. No money may be given with it, and the mother has also this month for reflection before finally resigning all claim. Here let it be added that not all these little ones are unfathered. Among those lately received are triplets, now five months old, whose mother, a working-man's wife, died after the births.

Addison House, the new Hostel, has been presented to the Society, with a handsome maintenance income, by Mrs. Creagh, who herself settles there to aid the work. So much interest has been aroused that Miss Andrew has been invited to Bournemouth, where an enthusiastic meeting promised developments, to Edinburgh, and also to Paris to help in the inauguration of schemes. A valuable feature in this work is the training of young girls in the temporary care of the children to become nursery nurses on the best modern lines. The Ministry of Health gives approval and aid to this excellent form of infant life-saving. It should be added that experience tends to show the desirability of taking a child as young as possible. It remains only for our Legislature to bring the law of inheritance by adopted children into at least as secure a position as is the case in other countries. As to the numbers dealt with, 6,000 cases have passed through the Association's offices, quite 50 per cent. being considered unsuitable. But after all sifting 1,000 cases are being dealt with at the head office, and it is expected that by Christmas 600 of these will be settled in families.

